# Transcranial Magnetic Stimulation Facilitates Neural Speech Decoding

**DOI:** 10.3390/brainsci14090895

**Published:** 2024-09-02

**Authors:** Lindy Comstock, Vinícius Rezende Carvalho, Claudia Lainscsek, Aria Fallah, Terrence J. Sejnowski

**Affiliations:** 1 Department of Psychiatry & Biobehavioral Sciences, UCLA, Los Angeles, CA 90095, USA; 2 Semel Institute for Neuroscience and Human Behavior, UCLA, Los Angeles, CA 90095, USA; 3 Postgraduate Program in Electrical Engineering, Federal University of Minas Gerais, Belo Horizonte 31270-901, MG, Brazil; 4 Computational Neurobiology Laboratory, The Salk Institute for Biological Studies, La Jolla, CA 92037, USA; 5 Institute for Neural Computation, UCSD, San Diego, CA 92093, USA; 6 Department of Neurosurgery, UCLA, Los Angeles, CA 90095, USA; 7 Division of Biological Sciences, UCSD, San Diego, CA 92093, USA

**Keywords:** neuromodulation, transcranial magnetic stimulation, phoneme discrimination, neural speech decoding, motor theory of speech perception

## Abstract

Transcranial magnetic stimulation (TMS) has been widely used to study the mechanisms that underlie motor output. Yet, the extent to which TMS acts upon the cortical neurons implicated in volitional motor commands and the focal limitations of TMS remain subject to debate. Previous research links TMS to improved subject performance in behavioral tasks, including a bias in phoneme discrimination. Our study replicates this result, which implies a causal relationship between electro-magnetic stimulation and psychomotor activity, and tests whether TMS-facilitated psychomotor activity recorded via electroencephalography (EEG) may thus serve as a superior input for neural decoding. First, we illustrate that site-specific TMS elicits a double dissociation in discrimination ability for two phoneme categories. Next, we perform a classification analysis on the EEG signals recorded during TMS and find a dissociation between the stimulation site and decoding accuracy that parallels the behavioral results. We observe weak to moderate evidence for the alternative hypothesis in a Bayesian analysis of group means, with more robust results upon stimulation to a brain region governing multiple phoneme features. Overall, task accuracy was a significant predictor of decoding accuracy for phoneme categories (F(1,135) = 11.51, *p* < 0.0009) and individual phonemes (F(1,119) = 13.56, *p* < 0.0003), providing new evidence for a causal link between TMS, neural function, and behavior.

## 1. Introduction

Transcranial magnetic stimulation (TMS) has been championed as an alternative method to indirect or correlational analyses [1,2]. The electromagnetic stimulation of cortical neurons creates an electrical field that depolarizes the membrane potential and pushes neurons past an excitation threshold. TMS is, therefore, argued to exert a causal effect that may induce a behavioral change [1,2]. At the same time, the effect of TMS is known to be complex. Depending on the stimulation threshold [3], frequency [3], duration [2], and coil orientation [4], affected neurons may be horizontally aligned interneurons [5] or the cortical columns of corticospinal neurons [6]. A synchronized or consistent response may be limited to neurons with axons of a certain length and position relative to the stimulating coil [7,8,9]. Despite the widespread application of TMS to study causal relations, the complex relationship between electromagnetic stimulation and its effect on cortical function has fostered debate about the extent to which TMS may influence neurons involved in volitional motor commands [1,10,11]. A measurable effect reflects the summation of post-synaptic potentials of opposite charges [12,13,14], which give rise to motor-evoked potentials (MEPs) [15,16,17], TMS-evoked potentials (TEPs) [18,19], neural oscillations, and connectivity changes [20,21]. As a result, stimulation parameters must be calibrated precisely to ensure that the net effect will inhibit or facilitate cortical excitability, and local and distal neuronal responses may interfere with the site-specific effect of interest [2,22].

TMS studies that aim to perturb linguistic processes often investigate the relationship between motor and perceptual neural circuits (for a review, see [11,23]), building on findings in the neuroimaging literature [24,25] that describe an interrelation between motor and perceptual circuits. In these TMS studies, the stimulation of motor cortex regions involved in speech perception has been shown to increase cortical excitability, as observed in the amplitude of the MEPs elicited in single-pulse paradigms [26,27,28,29] or increased effective connectivity in double-pulse paradigms [30]. Notably, ref. [31] illustrated that the higher cortical excitability induced via TMS may lead to a measurable behavioral and perceptual outcome that is related to the site-specific function of the TMS target. The authors created a double dissociation in phoneme discrimination ability and task reaction times by targeting cortical regions uniquely involved in the articulation of two categories of phonemes. The authors concluded that the higher excitability of neurons stimulated via TMS led to faster reaction times and a perceptual bias towards one of the two linguistic representations. Therefore, we oriented our study to the stimulation parameters and experimental design of [31], in which TMS produced a measurable and predictable effect on psychomotor activity.

The present study builds upon this literature to further investigate the nature of TMS effects and the limits of focal stimulation by means of neural speech decoding. Neural speech decoding utilizes neural activity thought to be associated with language as inputs for a classifier designed to detect a linguistic feature of interest. Frequently, these inputs are recorded from the motor cortex [32,33]. The increased cortical excitability induced via TMS could augment this activity in a task-relevant manner or induce noise that interferes with the decoding analysis. We assess whether the neural signals recorded during TMS will yield superior inputs for neural speech decoding, which would imply that task-relevant TMS augments the signal of interest over other induced effects. Secondly, we observe whether phonemes with overlapping cortical representations may be effectively targeted through the paradigm. To this end, we first replicate the findings of [31] and then show the same double dissociation between phoneme categories in a speech-decoding classification analysis performed on the neural data that were collected during the behavioral task.

**Figure 1:**
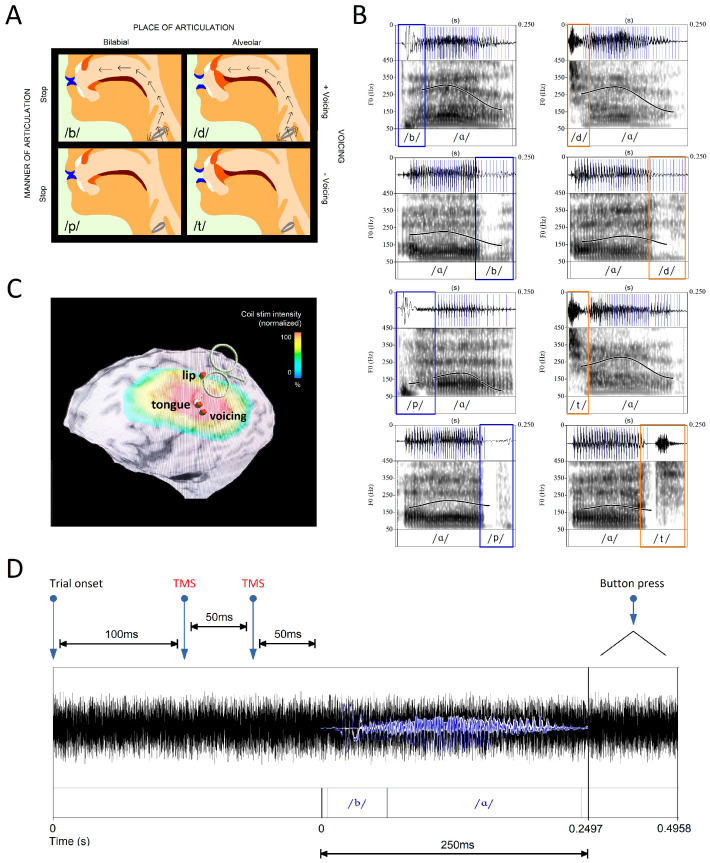
Phoneme Classes and Their Cortical Representation. (**A**) The phonemes included in this study (/b/, /p/, /d/, /t/) differ according to the place in the oral cavity where they are articulated (bilabial and alveolar—columns) and the degree to which they involve vocal cord movement (voiced and unvoiced—rows). (**B**) Vocal cord vibrations, represented by blue lines overlying the waveform, are generated via the phonemes /b/ and /d/ (waveforms are taken from the audio stimuli). After vowels, which are always voiced, vibrations perseverate into post-vocalic consonants. Differences in the waveforms and the degree of preseveration are observable among bilabial (blue) and alveolar (orange) phonemes. (**C**) The experimental paradigm stimulates sites in the motor cortex associated with phoneme articulation. Each site was taken from neuroimaging studies that reported the mean MNI coordinates corresponding to the peak motor cortex activation probability during a specific articulatory process (lip: −56, −8, 46; tongue: −60, −10, 25; voicing: −60, −15, 18) [35,36]. The site associated with voicing is adjacent to the tongue target and receives the same maximum stimulation intensity from the TMS coil. (**D**) Participants listened to stimuli items immersed in 500 ms of white noise to avoid performance at the ceiling in the phoneme discrimination task. Two TMS pulses were administered 50 ms prior to the phoneme onset with a 50-ms inter-pulse interval to replicate the excitatory paradigm in our reference study [31].

The experimental paradigm in [31] capitalizes on the properties of motor articulation to manipulate error rates in a phoneme discrimination task. To generate phonemes, neuromotor commands are sent to articulatory muscles that modify the airflow within the vocal tract [34]. Consonant phonemes are described by the muscle movements that produce them: (i) manner, which refers to how speech organs modify airflow; (ii) place, which indicates where airflow modification occurs in the vocal tract; and (iii) voicing, which describes the vibration of the vocal folds. Each consonant phoneme comprises a unique combination of these articulatory properties (Figure 1A). Phonemes within the set of bilabial and alveolar stops (/b/, /p/, /d/, /t/) share a manner of articulation (oral stops), but they differ according to whether they are primarily produced using the lips (bilabial—left column) or tongue (alveolar—right column), yielding two phoneme categories that can be distinguished by the location and method of motor articulation. The phonemes are further distinguished by those produced with vibration of the vocal folds (top row) or without (bottom row).

The task paradigm (Figure 1D) requires a button-press identification of sound files comprising alveolar (/d/, /t/) or bilabial (/b/, /p/) stops. The authors illustrated that the stimulation of the motor cortex via two single TMS pulses administered in rapid succession can influence which phoneme category is perceived. TMS administered to cortical regions governing either lip or tongue movements (Figure 1C) produced a significant effect on performance: the response accuracy and speed increased in trials of phonemes articulated with muscles controlled via the stimulated region and declined in trials of phonemes controlled using muscles associated with the unstimulated region. The findings support a TMS-induced bias in perception resulting from the stimulation of motor cortex neurons.

This approach, which stimulates phonemes on the category level, likely reflects the challenges of focal stimulation with TMS coils [37]. Theoretically, individual phonemes may be targeted if regions associated with two articulatory features are stimulated concurrently. Only one phoneme in each category of the set (/b/, /p/, /d/, /t/) is produced with vocal-fold vibration (Figure 1A). Vowels generate sustained vibration, such that their voicing properties will perseverate, greatly enhancing the extent of vibration that accompanies the following consonant phoneme (Figure 1B). While lip and tongue articulation have been localized to non-adjacent regions of the motor cortex, voicing is controlled by a region in close proximity to the site of tongue articulation [35,36] (Figure 1C). The resulting overlap in stimulation across tongue and voicing sites should produce a graded effect across the four phonemes, depending on their specific properties. Thus, the stimulation of multiple features in partially overlapping feature sets could allow for more precise targeting.

Confirmation that electromagnetic stimulation of the motor cortex, as implemented in our paradigm, elicited the same task-relevant behavioral response observed in [31] is a necessary first step prior to evaluating whether TMS-induced cortical excitability will improve the suitability of EEG signals for speech decoding. First, we replicated the previously reported bias in phoneme-category discrimination through two separate experiments, and we evaluated evidence of a graded effect for individual phoneme discrimination ability. Next, we performed a classification of the EEG data collected during the phoneme discrimination task to determine whether the stimulation of each cortical region will result in a more accurate prediction of the associated phoneme.

## 2. Methods and Materials

### 2.1. Subject Details and Inclusion Criteria

Participants (aged 20–40) were recruited from the UCLA campus by means of flyers. All participants possessed no diagnosis of any neurological, psychiatric, or developmental disorders, self-reported normal hearing, and no contraindications for TMS or MRI protocols (e.g., implanted medical devices, implanted metal, pregnancy, a personal or family history of seizures, and exclusionary medications). The initial screening required the completion of an abbreviated version of the experimental task to ensure that participants understood the task directions and could successfully discriminate between phonemes. Left-hemisphere lateralization of the language-processing regions in all participants was established during an fMRI scan in which the participants performed the discrimination task. Ten participants (6 female) were recruited in 2019. This participant number was established based on the sample size of our reference study [31]. Two individuals (1 female) were excluded from the 2019 data set due to modifications made to the stimulus audio files after their participation. All ten participants contributed neural and behavioral data, which were collected during the discrimination task. Twenty participants (10 female) were recruited for the second experiment in 2021. One participant (male) was excluded due to poor task performance, and three participants (male) were excluded due to complications with the TMS equipment that may have led to imprecise targeting. All participants contributed neural data. Only the final ten participants recorded their responses during the discrimination task. All participants provided informed consent and were paid for the two experimental sessions.

### 2.2. Experimental Design and Data Collection

**Figure 2:**
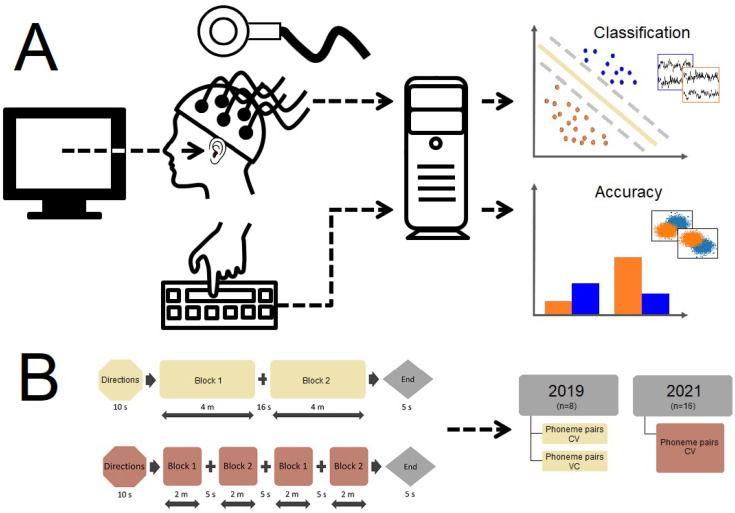
Experimental Paradigm. (**A**) Participants listened to phoneme stimuli presented via computer-based experiment-presentation software. Concurrently, EEG signals were recorded as participants identified the phoneme they heard with a button-press response input on a computer keyboard. The task was performed with TMS under experimental conditions and without TMS under the control condition. After data collection, a classification analysis was conducted on the EEG signals, and accuracy was computed for the aggregate task-response data. (**B**) The task was administered in two blocks in 2019 and in four blocks in 2021; both CV and VC phoneme pairs were presented in 2019, and only CV phoneme pairs were presented in 2021. The presentation order of blocks and stimuli lists was counterbalanced across participants. EEG data were obtained for 8 participants in 2019 and 16 participants in 2021. Task-response data were obtained from 8 participants in 2019 and 2021.

The study was conducted in three sessions. First, the participants underwent an intake interview to ensure that they met the study inclusion criteria and possessed no contraindications. Participants who performed an abbreviated phoneme discrimination task with at least 75% accuracy were enrolled. Second, the participants underwent an MRI scan to aid in neuronavigation for the TMS procedure, during which they performed the discrimination task in the MRI scanner to lateralize their language processing areas. Finally, EEG signals, button-press responses, and reaction-time data were recorded while the participants performed the phoneme discrimination task. TMS was targeted to areas of the motor cortex associated with the production of the designated phonemes (Figure 2).

### 2.3. MRI Scanning

The scanning protocol was conducted at the UCLA Center for Cognitive Neuroscience with a Siemens Prisma-FIT 3T Scanner. The participants were provided with ear protectors and headphones to reduce the scanner noise from 45 to 60 dB, thus ensuring that the stimuli could be heard clearly and that the noise level was not uncomfortably loud. The participants were asked to lie motionlessly during scanning. High-resolution anatomical images were acquired, followed by a functional scan in which the participants were directed to either relax passively while looking at a fixation cross or perform a right-handed button-press phoneme discrimination task. The functional data were acquired in a block design with a TR of 800 ms and a BOLD-weighted echoplanar imaging sequence aligned in parallel to the bicommissural plane, yielding 36 slices covering the whole brain. Each slice was 3-mm thick with a 1-mm gap between slices, and each slice was acquired as a 64-×-64 matrix yielding an in-plane resolution of 2 × 2 mm. The total duration of the scan was 40 min. fMRI data were analyzed in FSL (version 5.0.10). The data were corrected for motion artifacts for each participant and transformed into a common brain space according to the MNI (Montreal Neurological Institute) template. The data were resampled into 2-mm isotropic voxels and smoothed with a 4-mm Gaussian filter. Effects were estimated using a general linear model (GLM). The protocol was not intended to isolate distinct brain areas for each phoneme or phoneme category on the level of individual participants. Instead, a subtraction analysis was performed between the listening task (all phonemes) and rest (the silent observation of a fixation cross). The functional maps exhibited bilateral fMRI activation over our region of interest—the motor cortex—with a greater extent of activation in the left hemisphere.

### 2.4. TMS During EEG Recording

The TMS-EEG protocol was conducted at the Neuromodulation Division of the Semel Institute for Neuroscience and Human Behavior at UCLA. The TMS equipment included a Magstim Super Rapid Plus1 stimulator and a figure-of-eight 40-mm coil. The EEG system included an eego^TM^ sports WaveGuard 64-channel EEG cap and eego mylab system compatible with electromagnetic stimulation. Targeting was completed using the Visor 2 neuronavigation system. The electrode positions were digitized and registered to individual participant MRIs using the ANT Neuro Xensor. EEG signals were bandpass-filtered 0.1–350 Hz, sampled at 2000 Hz, and referenced to the CPz electrode. All electrode impedances were kept <5 kΩ. The appropriate stimulation intensity for TMS studies is determined on an individual basis [38]. Prior to the experimental session and after the application of the EEG cap, the motor threshold (rMT) of each participant was determined by eliciting motor-evoked potentials (MEPs) in the first dorsal interosseus (FDI) muscle of the dominant hand at the minimum amount of stimulation needed to evoke an MEP in a hand muscle after a single pulse over M1. Single TMS pulses were delivered to locations in the motor cortex contralateral to the dominant hand. The intensity of the stimulation was gradually lowered until reaching a level of stimulator output at which 5 out of 10 MEPs in the hand muscle had an amplitude of at least 50 microvolts. In accordance with [31], stimulation was administered at 110% of the FDI rMT, and the coil was maintained at a 45° orientation to the interhemispheric fissure. The participants were provided with ear protectors [39], and a physician observed the motor-thresholding procedure to ensure that no negative effects were incurred by the participants. The range of rMT values fell within normal parameters [40].

TMS targeted areas of the motor cortex involved in lip and tongue movements [41]. The stimulation targets were defined as the MNI coordinates of peak motor cortex activation in LipM1 and TongueM1 during lip and tongue articulatory movements (lips: −56, −8, 46; tongue: −60, −10, 25), which were taken from the literature [36] and the reference study [31]. However, cortical functional localization shows considerable variation by individual [42] and task [43,44]. It was important to target regions associated with both phoneme articulation and the perception task. Therefore, the coordinates were overlaid over the activation map of the task results for each participant to ensure an overlap between the targets and individual task localization. The target was taken as the nearest peak to the MNI coordinate. TMS elicits a period of excitatory activation with an onset latency of 50–80 ms after stimulation [45]. We reproduced the design of [31] to ensure an excitatory neural response that would translate into task facilitation. Each trial delivered two TMS pulses separated by a short interpulse interval (50 ms) at one of the stimulation targets. The excitation of the cortical region not involved in stimulus production (i.e., TMS at LipM1 during alveolar phoneme presentation) may result in neural noise that interferes with the discrimination task. The audio stimulus followed 50 ms after the second TMS pulse. One target was stimulated per run (counterbalanced across participants).

### 2.5. Phoneme Discrimination Task

The participants were asked to identify speech sounds with a button-press response. The auditory stimuli were presented via laptop speakers: consonants (Cs) comprised four phonemes (/b/, /p/, /d/, /t/), and vowels (Vs) comprised five phonemes (/i/, /ɛ/, /A/, /u/, /oU/), yielding 40 phoneme pairs (20 CV/20 VC). The participants listened to audio clips immersed in 500 ms of white noise. This created a mild background distraction to ensure that participants did not perform the task at the ceiling so that we could measure the relative accuracy in phoneme discrimination across conditions. The participants were instructed to respond as fast as possible with a button press after they had identified the phoneme. In the case of multiple button presses, correct trials were calculated from the initial button press. Participants who exhibited a biased response strategy (i.e., failure to select from the full set of phonemes) were excluded. Stimulus presentation and the recording of reaction-time data were conducted in PsychoPy [46]. Two lists of stimuli were used, with each list assigned to one block. A run comprised all blocks administered sequentially during TMS to the same target. One run included a block of CV pairs, followed by a block of VC pairs. A 16-second break separated the blocks. A 5-minute break was provided between runs. The participants completed 120 trials in each run: 80 with TMS and 40 control trials. All phoneme pairs were presented in a pseudo-randomized order.

The reversal of the order of phonemes in VC stimulus pairs increases the time interval between the TMS pulse and the consonant. Because timing plays a key role in determining the ultimate effect of TMS, the protocol cannot be assumed to produce the same excitatory effect on VC stimuli; this condition may, therefore, serve as an interesting comparison. Upon confirmation in 2019 that the protocol produced a different behavioral response for VC pairs, only CV pairs were tested in 2021, using a slightly modified protocol. The same CV lists were split in two, such that four blocks were administered per run, with a 5-s break between blocks. The performance for the phoneme /d/ in the 2019 control condition was notably elevated relative to other phonemes. Therefore, in 2021, the decibel level of the white noise in which stimuli were immersed was increased to ensure that the control condition would prove uniformly challenging. All other aspects of the task remained the same. Minimal modifications to this procedure were made during the intake and scanning sessions. For the initial assessment, half of the task was performed. During fMRI data collection, the full-length task was administered. Stimuli were combined in blocks with a jittered 16-s ISI by articulation type (bilabial and alveolar) to achieve greater power.

Button-press tasks assume that participants will respond accurately; in practice, there is an element of motor coordination through which participants may press either a correct or incorrect button by accident (creating both type I and type II errors). We know from participant feedback that random buttons were periodically pressed, given the distractions of TMS. On occasion, participants were aware that they had pressed the wrong button upon having correctly heard the sound. Therefore, missed trials (i.e., when the participant did not press a button, having not perceived the sound) were excluded from the analyses, whereas neural data from all trials when the participant heard and responded to a sound (including incorrect trials) were retained in the decoding analyses. Because we know the task performance allowed for both type I and type II errors, we cannot assume that the inclusion of all trials reduced the decoding accuracy or that the exclusion of some portion of trials would have represented a more reliable decoding accuracy. There is no way to know the true ratio of response types, and we suspect a larger number of false negatives based on participant feedback, so we opted to retain all trials. An analysis limited to correct trials might lead to a false sense of certainty, as there are likely some false positives included in the task results, and participants who performed well would be over-represented in the data set. Moreover, the ability to correctly perceive a sound and the ability to correctly perform a button-press task are not strictly equivalent. Generally speaking, we assume that the error of either type is low because we screened for participants who could perform the task at greater than 75% accuracy prior to initiating TMS trials.

### 2.6. Quantification and Statistical Analyses

The protocol required sustained attention during a lengthy period of TMS. The mean reaction time and standard deviation were calculated to confirm that the participants were attentive to the task throughout the procedure. These metrics are documented in .csv files uploaded to the data repository. In the 2019 data set, some variation in trial numbers was observed due to the exclusion of missed trials in which no response was recorded or due to rotation in the list of stimuli administered to each participant. All excluded trials resulted from missed trials. No subjects performed a button-press decision for less than 90% of the total list, with the exception of P04 in the VC condition with LipTMS. In the 2021 data set, all trials were uploaded irrespective of a button-press response.

Bayesian analyses were performed using JASP [47], and linear mixed-effects models were performed in RStudio [48] with the lmerTest package [49]. All statistical analyses are publicly available at https://osf.io/e82p9/(accessed on 2 August 2024). We implemented the Bayesian *t*-test framework proposed by [50,51] on the behavioral task data. Task accuracy is measured as the average hit rate for each phoneme category or individual phoneme in the experimental condition minus the average hit rate in a matched control condition. Due to potential site-specific effects, control trials recorded during each type of stimulation (lip motor cortex—LipM1; tongue motor cortex—TongM1) were averaged separately, yielding two sham conditions (e.g., LipM1 TMS average accuracy − LipM1 sham average accuracy = relative task accuracy). A Bayesian approach may be more informative for small-N studies because the credible intervals do not depend on large-N approximations [52]. Instead, unlike p-values, Bayes factors assess the degree of evidence for the alternative hypothesis along a continuum. Such analyses provide a three-way distinction between the null hypothesis, the alternative hypothesis, and no evidence. This is important because a highly powered study may yield a significant result from data that are insufficiently sensitive to illustrate an effect or fail to show a significant result in low-powered studies that do yield sensitive data; power does not guarantee that we can distinguish between these three states of evidentiality [53].

Average decoding accuracy was calculated from EEG data recorded across trials by applying our signal-processing technique to triplets” of nearby channels simultaneously as a form of data augmentation, in the same way as described for the computation of ERPs in [54]. A transformation of the data was first performed to allow for the data frames to be compared. In decoding analyses, random under-sampling was used to equalize the number of trials per condition. Cross-validation was performed with k-fold random subsampling. As a result, we obtained a smaller data set and near-zero error in the random subject variable. This difficulty is commonly encountered in psychological experiments; best practice recommends that the analysis still be considered appropriate when near-zero error is the source of a singular fit in the model [55].

### 2.7. Delay Differential Analysis

We employed a novel signal-processing technique, delay differential analysis (DDA), as part of the neural decoding classification analyses. The technique combines differential embeddings with linear and nonlinear nonuniform functional delay embeddings. The integration of nonlinear dynamics allows information from the data to be detected that may not be observable in traditional linear methods. DDA requires minimal pre-processing of the EEG data, which eliminates a highly subjective step in the data analysis. Sparse DDA models have several advantages over the high dimensional feature spaces of other signal-processing techniques: (i) the risk of overfitting is greatly reduced; (ii) the sparse model concentrates on the overall dynamics of the system and cannot additionally model noise; (iii) DDA is computationally fast; and (iv) there is no need for pre-processing (i.e., the removal of motion artifacts like eye blinks, etc.), except the normalization to zero mean and unit variance for each data window, in order to ignore amplitude information and concentrate on the system dynamics. DDA is a detection/classification technique that aims not to model the data (i.e., reconstruct the equations for the underlying process generating the data) but to distinguish between data classes. The classification performance of the DDA models is assessed by the area under the receiver operating characteristic.

The term “DDA models” may refer to the DDA model structure, as well as the delays within the model structure. The model structure was fixed throughout our analyses, and therefore, the complexity of the model did not change: it was the delays that were determined for a specific classification problem. DDA models (i.e., the model structure) can be seen as sparse Volterra-series models with only three terms, two delays, and a quadratic order of nonlinearity. The most general DDA model is

(1) $$\dot{x}=\sum_{i=1}^{I}a_{i}\prod_{n=1}^{N}x_{\tau_{n}}^{m_{n,i}}\;.$$

for $\tau_{n}\in N$, $m_{n,i}\in N_{0}$ (N is the set of positive natural numbers, and $N_{0}$ is the set of natural numbers). *N* is the number of delays (usually 2), *I* is the number of terms (typically around 3), and $x_{\tau_{n}}=x\left(t-\tau_{n}\right)$, relating the signal derivative $\dot{x}$ to the signal non-uniformly shifted in time. We then used the coefficients $a_{i}$ and the least square error $\rho_{u}$ as features. Most of the terms in Equation (1) were set to zero in the model-selection step of the analysis using random subsampling cross-validation.

Finding the best model can be done supervised or unsupervised. In [54], we selected the best model for the epileptic seizure analysis of iEEG data from all models with three terms and up to quartic nonlinearity using a genetic algorithm. This was done unsupervised by finding the DDA model, as well as the delays that have the lowest least square error of randomly selected data segments before and after seizure onset, as determined by a neurologist. Interestingly, most of the models found had two linear terms and one nonlinear term. This class of models was found again when we performed a supervised, exhaustive search on EEG data from a large data set of 877 schizophrenia patients and 753 nonpsychiatric comparison subjects who underwent mismatch negativity testing [56] in [57], where we built a spindle detector from iEEG data, and other EEG studies. For other data classes, such as heart electrocardiogram data, other models were found [58].

The DDA model structure consists of two sets of parameters: (i) the delays and model form are fixed parameters that are kept constant throughout the analysis; and (ii) the coefficients $\left(a_{1},a_{2},a_{3}\right)$ and the fitting error of the model are free parameters. The nonlinearity of the terms or the complexity of the DDA model depends on the data class. For EEG data, a model with two linear terms and one nonlinear term has been shown to act as a good model in a variety of studies [54,56]. The coefficients are used as features to distinguish different dynamics in the data. The DDA model for EEG data used here is

(2) $$\dot{x}=a_{1}x_{1}+a_{2}x_{2}+a_{3}x_{1}^{2}\;$$

where $x_{i}=x\left(t-\tau_{i}\right)$ is the signal delayed due to $\tau_{i}\in N$, and the fixed parameters are the same as in [56,59]. We found that one of the free parameters, namely $a_{3}$, can be used to describe neural activity in a manner similar to ERPs, although they are not the same phenomenon (see [56]). In most cases, there is no direct relation between frequencies and any of the model parameters, as explained in [60]. For a model with only linear terms, a direct connection to spectral analysis can be found; as soon as nonlinear terms are added to the model, each coefficient of the model corresponds to a combination of higher-order statistical moments (see [60]). In the analyses performed here, the delays were $\tau_{1}=6\;\delta t$ and $\tau_{2}=16\;\delta t$, with $\delta t=\frac{1}{f_{s}}$, and the sampling rate was $f_{s}=2000$ Hz (double the delays in [56] because the sampling rate was doubled). The window length was 30 ms, and the window shift was 1 ms. To explain how to use the same DDA model for data with double the sampling rate, we use the same approach as explained in [57]. For a time series, x(t), of length *L*, Equation (2) can be rewritten as a matrix equation in the following way:

(3) $$\begin{array}{cccc} \left(\begin{array}{c} \dot{x}(t+1)\\ \dot{x}(t+2)\\ \dot{x}(t+3)\\ \vdots \\ \dot{x}(t+L) \end{array}\right) & = & \left(\begin{array}{ccc} x(t+1-\tau_{1}) & x(t+1-\tau_{2}) & x{\left(t+1-\tau_{1}\right)}^{2}\\ x(t+2-\tau_{1}) & x(t+2-\tau_{2}) & x{\left(t+2-\tau_{1}\right)}^{2}\\ x(t+3-\tau_{1}) & x(t+3-\tau_{2}) & x{\left(t+3-\tau_{1}\right)}^{2}\\ \; & \vdots \\ x(t+L-\tau_{1}) & x(t+L-\tau_{2}) & x{\left(t+L-\tau_{1}\right)}^{2} \end{array}\right) & \left(\begin{array}{c} a_{1}\\ a_{2}\\ a_{3} \end{array}\right) \end{array}\;\dot{x}=M_{x}\;A$$

Note that $M_{x}$ is an (L×3) matrix. *L* is the number of data points for each window for the estimation of the three free parameters, $a_{1,2,3}$. For data with double the sampling rate, we rewrote Equation (3) in the following way:

(4) $$\begin{array}{ccc} \left(\begin{array}{c} {\dot{x}}_{2j-1}\\ {\dot{x}}_{2j} \end{array}\right)\; & = & \left(\begin{array}{c} M_{x_{2j-1}}\\ M_{x_{2j}} \end{array}\right)\;A \end{array}$$

where

(5) $$\begin{array}{c} {\dot{x}}_{2j-1}=\frac{d}{dt}\left(\begin{array}{c} x(t+1)\\ x(t+3)\\ x(t+5)\\ \vdots \\ x(t+(2L-1)) \end{array}\right)\;,\;{\dot{x}}_{2j}=\frac{d}{dt}\left(\begin{array}{c} x(t+2)\\ x(t+4)\\ x(t+6)\\ \vdots \\ x(t+(2L)) \end{array}\right) \end{array}$$

are the numerical derivatives, and

(6) Mx2j−1=x(t+1−2τ1)x(t+1−2τ2)x(t+1−2τ1)2x(t+3−2τ1)x(t+3−2τ2)x(t+3−2τ1)2x(t+5−2τ1)x(t+5−2τ2)x(t+5−2τ1)2⋮x(t+(2L−1)−2τ1)x(t+(L−1)−2τ2)x(t+(2L−1)−2τ1)2 Mx2j=x(t+2−2τ1)x(t+2−2τ2)x(t+2−2τ1)2x(t+4−2τ1)x(t+4−2τ2)x(t+4−2τ1)2x(t+6−2τ1)x(t+6−2τ2)x(t+6−2τ1)2⋮x(t+(2L)−2τ1)x(t+(2L)−2τ2)x(t+(2L)−2τ1)2

are the matrices with alternating data points and the double delays. This way, the delays are doubled in relation to Equation (3).

The classification task was done in a subject-dependent manner, with one model by participant and TMS condition. Non-overlapping triples of neighboring channels were combined, resulting in 20 channel triples. Models were obtained with dynamical ergodicity DDA (DE-DDA) applied to these triples on sliding windows of 30 ms and window shifts of 1 ms. From these, mean and standard deviation values were taken for each trial, resulting in the features that serve as inputs to the classifiers. Classification of phonemes was achieved with SVD, as described in [61]. Leveraging the spatiotemporal aspects of the data, training of the SVD classifier was done through non-connected time trials and testing with time-connected trials.

## 3. Results

### 3.1. Phoneme Discrimination Task

Two experiments utilizing the same protocol were performed in 2019 and 2021. After the exclusion of problematic data (see Section 2), an equal number of participants in 2019 (*n* = 8) and 2021 (*n* = 8) contributed data from the behavioral task.

#### 3.1.1. Phoneme Categories

We predicted that the stimulation of the region controlling lip-muscle movements would increase the discrimination accuracy for bilabial consonants and that stimulation of the region controlling tongue-muscle movements would increase this accuracy for alveolar consonants. At the same time, we predicted that error rates would increase for phonemes not associated with the stimulation sites. The 2019 experiment compared the results for consonant–vowel (CV) and vowel–consonant (VC) pairs to confirm that the protocol design affected consonants in the pair-initial position. Only CV pairs indicated support for the alternative hypothesis. Therefore, only CV pairs were analyzed in the 2021 experiment.

Within this paradigm, the null hypothesis postulated that there would be no category-specific difference in task accuracy when phoneme discrimination was performed during task-relevant TMS versus when the task was performed without TMS; moreover, any difference should reflect a greater relative accuracy when the TMS target corresponds to the associated phoneme category (i.e., /b/, /p/ during LipM1 TMS and /d/, /t/ during TongM1 TMS). The one-sided alternative hypothesis stated that a greater relative accuracy would be obtained when the TMS target corresponded to the phoneme category produced via the articulators governed by this brain region. Importantly, relative accuracy was measured because it is inherently easier to perform the discrimination task during the sham condition. Greater noise and scalp sensations were reported during experimental trials.

The Bayes factor provides a continuous measure of evidence for $H_{+}$ over $H_{0}$. The data were equally well predicted by both models when the Bayes factor was 1. As the Bayes factor increased above 1, the evidence favored $H_{+}$ over $H_{0}$; the reverse was true as the Bayes factor decreased below 1. A Bayes factor of 3 is often considered to be the amount of evidence that approximates a *p*-value of 0.05 [50,62]. However, this is an arbitrary level selected to correspond to a commonly used yet problematic metric [63]; other authors recommend establishing a threshold for each specific case [64]. Our brief report considers evidence from a relatively small data set. Thus, here, we are primarily interested in which category of evidentiality is supported, whether evidence for a hypothesis trends in the same direction across studies, and under which conditions the evidence may be more robust.

Table 1 shows that the Bayes factor indicated minimal evidence for $H_{+}$ in all CV conditions of the 2019 and 2021 experiments and approached moderate evidence for $H_{+}$ in the 2021 alveolar condition. In contrast, the Bayes factor indicated moderate evidence for $H_{0}$ in the bilabial condition and no evidence for $H_{+}$ in the alveolar condition. The BF_+0_ for CV items equaled between 1.188 and 2.002, which means the data were between approximately 1.188 and 2.002 times more likely, depending on the condition, to occur under $H_{+}$ than $H_{0}$. The BF_+0_ for VC items equaled only between 0.121 and 0.807, which means the data were between approximately 0.121 and 0.807 times more likely, depending on the condition, to occur under $H_{+}$ than $H_{0}$. The error percentages were small, which indicates that the algorithm used to obtain the results has stability; the error percentage was larger in the inconclusive alveolar VC condition. Notably, the Bayes factor was consistently larger for alveolar phonemes in both the CV and VC trials.

**Figure 3:**
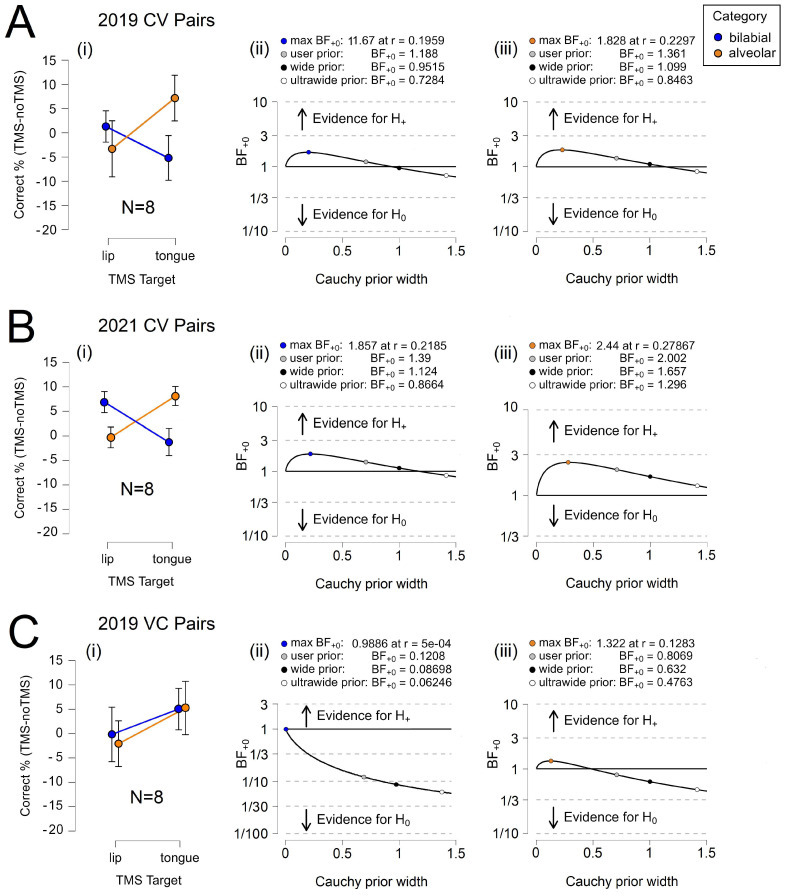
Phoneme Category Discrimination. (**A**) 2019 CV results for (**i**) relative accuracy, and parameter estimation for (**ii**) bilabial phonemes and (**iii**) alveolar phonemes. (**B**) 2021 CV results for (**i**) relative accuracy, and parameter estimation for (**ii**) bilabial phonemes and (**iii**) alveolar phonemes. (**C**) 2019 VC results for (**i**) relative accuracy and parameter estimation for (**ii**) bilabial phonemes and (**iii**) alveolar phonemes. Error bars represent the 95% confidence intervals.

Figure 3 illustrates relative task accuracy and the results for parameter estimation. For the CV trials, we observed a clear double dissociation between the phoneme categories in 2019 and 2021 yet no double dissociation in the VC trials. Interestingly, TongM1 TMS appeared to elicit a marginal effect in the VC trials. The robustness of the Bayes factor to our prior specification is shown in BF_+0_ as a function of the prior width, *r*. When the Bayes factors for the user prior was over 1.3, the Bayes factor appeared to be relatively stable across several Cauchy prior widths. The accuracy results of the 2019 /d/ control task were abnormally high relative to those of the other phonemes; as a result, when /d/ and /t/ are graphed together, the error rates misleadingly appear to converge during TongM1 TMS. Here, /d/ is excluded from the line graph but not the analysis. We present the full set of data in Supplementary Material.

#### 3.1.2. Individual Phonemes

We evaluated whether the effects observed at the category level would persist for individual phonemes. In particular, we considered whether individual phonemes would show a graded effect across TMS targets due to the overlap in stimulation between the TongM1 target and a brain region governing the voicing of phonemes (see Figure 1C). Here, we analyze the 2021 CV data, for which a more ideal decibel level for white noise had been set, to allow a consideration of all four phonemes. Table 2 shows moderate evidence for $H_{+}$ in the /b/ and /t/ CV conditions. The BF_+0_ equaled between 2.010 and 3.255, indicating that the data were between 2.010 and 3.255 times more likely to occur under $H_{+}$ than $H_{0}$. In contrast, the Bayes factor indicated no evidence for $H_{+}$ in the /p/ and /d/ CV conditions, where the BF_+0_ equaled between 0.596 and 0.646, and the data were between 0.596 and 0.646 times more likely to occur under $H_{+}$ than $H_{0}$. The error percentages were consistently small, which demonstrates that the algorithm used to obtain the results has stability.

The VC conditions revealed a very different picture. The Bayes factor indicated minimal evidence for $H_{0}$ in the /p/ and /t/ VC conditions and no evidence for $H_{+}$ in the /b/ VC condition. The BF_+0_ equaled between 0.336 and 0.344 for /t/ and /p/, respectively, and the data were 0.336 and 0.344 times more likely to occur under $H_{1}$ than $H_{0}$. The BF_+0_ for /b/ equaled 0.959, and the data were 0.959 times more likely to occur under $H_{1}$ than $H_{0}$. These conditions possess a high error percentage, suggesting that our model does not describe the data efficiently; this confirms our assumption regarding the timing of the protocol for targeting the initial phoneme in phoneme pairs. However, surprisingly, the Bayes factor did indicate minimal evidence for $H_{+}$ in the /d/ VC condition with a small error percentage. The BF_+0_ equaled 1.385, and the data were 1.385 times more likely to occur under $H_{+}$ than $H_{0}$. This may indicate some evidence of an effect related to the voicing feature: /d/ is the only phoneme described by both tongue articulation and voicing.

**Figure 4:**
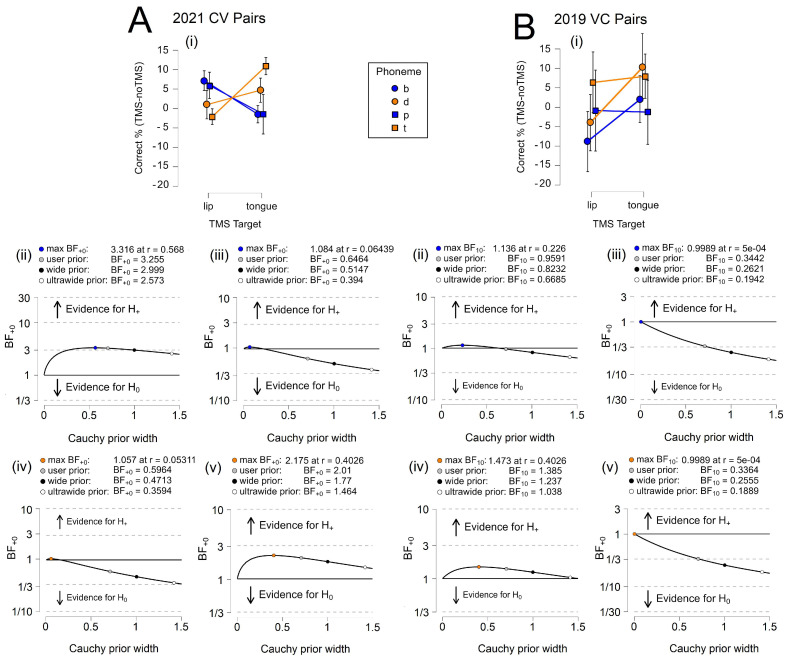
Individual Phoneme Discrimination. (**A**) 2021 CV results for (**i**) relative accuracy, and robustness parameters for (**ii**) /b/, (**iii**) /p/, (**iv**) /d/, and (**v**) /t/. (**B**) 2019 VC results for (**i**) relative accuracy, and robustness parameters for (**ii**) /b/, (**iii**) /p/, (**iv**) /d/, and (**v**) /t/. Error bars represent the 95% confidence intervals.

Figure 4 illustrates relative task accuracy and the results for parameter estimation. For the CV trials, we observed a double dissociation between phonemes in 2021. There was considerable error in the VC condition, making it difficult to draw conclusions. As anticipated, a dissociation between bilabial and alveolar phonemes was absent in the VC trials. Again, TongM1 TMS appeared to elicit a marginal effect. The robustness of the Bayes factor to our prior specification is shown in BF_+0_ as a function of the prior width, *r*. The Bayes factors for the user prior appeared to be relatively stable across several Cauchy prior widths when over 1.3.

In summary, the results of the behavioral trials confirm that the protocol was correctly designed to influence the perception of the initial consonant in phoneme pairs. The performance trended towards a more accurate perception of alveolar phonemes during TongM1 TMS. Upon disambiguation, we see an increased ability to discriminate the unvoiced alveolar phoneme /t/ in CV pairs during TongM1 TMS. This is not the case for /d/; however, as noted, some discrepancies emerged regarding the perceptibility of /d/ in white noise relative to the other phonemes. Secondly, the increased ability to discriminate voiced phonemes during TongM1 TMS appears as a trend that might be substantiated with the analysis of a larger data set. The findings are consistent with an additional effect of voicing caused by the overlap in stimulation across the cortical regions involved in tongue articulation and voicing. We may conclude that not all phonemes are equally affected by the protocol.

### 3.2. Neural Speech Decoding

In the behavioral task, TMS may have either increased the hit rate for true positives when the associated target was stimulated or increased the miss rate for false positives when a non-associated target was stimulated, thus biasing the participant towards the selection of an incorrect phoneme or phoneme category. Given the fact that we measured relative accuracy and that these metrics fluctuated across different phonemes in the control condition, it is difficult to ascertain which of these scenarios is more likely. Therefore, we investigated whether the TMS protocol would induce a facilitatory effect on the motor neurons governing phoneme articulation, which would be measurable from the EEG signals. Specifically, we sought to determine whether the stimulation of task-specific motor regions would result in superior inputs for neural speech decoding, which could be interpreted as support for the facilitation hypothesis. Classification analyses were conducted on the EEG signals collected during the phoneme discrimination task performed in 2019 (*n* = 8) and 2021 (*n* = 16). Neural data were collected from the participants who contributed the behavioral data analyzed above. In 2021, an additional cohort of participants contributed neural data recorded during the discrimination task, leading to a two-fold increase in neural data only.

#### 3.2.1. Phoneme Categories

We predicted that the stimulation of the region controlling lip-muscle movements would increase the classification accuracy of bilabial consonants, whereas the stimulation of the region controlling tongue-muscle movements would increase the classification accuracy of alveolar consonants. At the same time, we predicted that the decoding accuracy would decrease for phonemes that were not associated with the stimulation sites. We anticipated that the findings would illustrate a double dissociation that closely corresponded to the one observed in the behavioral analyses. Again, we considered both CV and VC pairs from the 2019 experiment and CV pairs only from the 2021 experiment.

Within this paradigm, the null hypothesis postulated that there would be no category-specific difference in decoding accuracy when phoneme discrimination was performed during task-relevant TMS versus when the task was performed without TMS; moreover, any difference should reflect a greater relative accuracy when the TMS target corresponded to the associated phoneme category. The one-sided alternative hypothesis stated that a greater relative decoding accuracy would be obtained when the TMS target corresponded to the phoneme category produced via the articulators governed by this brain region. As noted, the sham condition was inherently easier: effortful processing in TMS conditions may augment the neural signal (see [25]), or any real effect may be diminished, as postulated for the behavioral-task results.

Table 3 shows that the Bayes factor indicates moderate evidence for $H_{0}$ in the alveolar CV condition in 2019 and both VC conditions in 2019. The BF_+0_ equaled 0.307 and 0.344 for alveolar phonemes in the 2019 CV condition and between 0.157 and 0.196 in the 2019 VC conditions. Thus, the data were 0.307 and between 0.157 and 0.196 times more likely to occur under $H_{1}$ than $H_{0}$, respectively. However, the error percentage was only small for bilabial phonemes in the 2019 VC condition, suggesting that our model may not effectively describe the alveolar data. The Bayes factor indicates no evidence for $H_{1}$ in either the 2019 or 2021 CV bilabial conditions. The BF_+0_ equaled between 0.444 and 0.495, respectively, and the data were 0.444 and 0.495 times more likely to occur under $H_{1}$ than $H_{0}$, with small error percentages. Yet, the Bayes factor did indicate moderate evidence for $H_{1}$ in the alveolar CV condition of 2021. The BF_+0_ equaled 3.166, the data were 3.166 times more likely to occur under $H_{1}$ than $H_{0}$, and the error percentage was small, suggesting that this model is stable.

**Figure 5:**
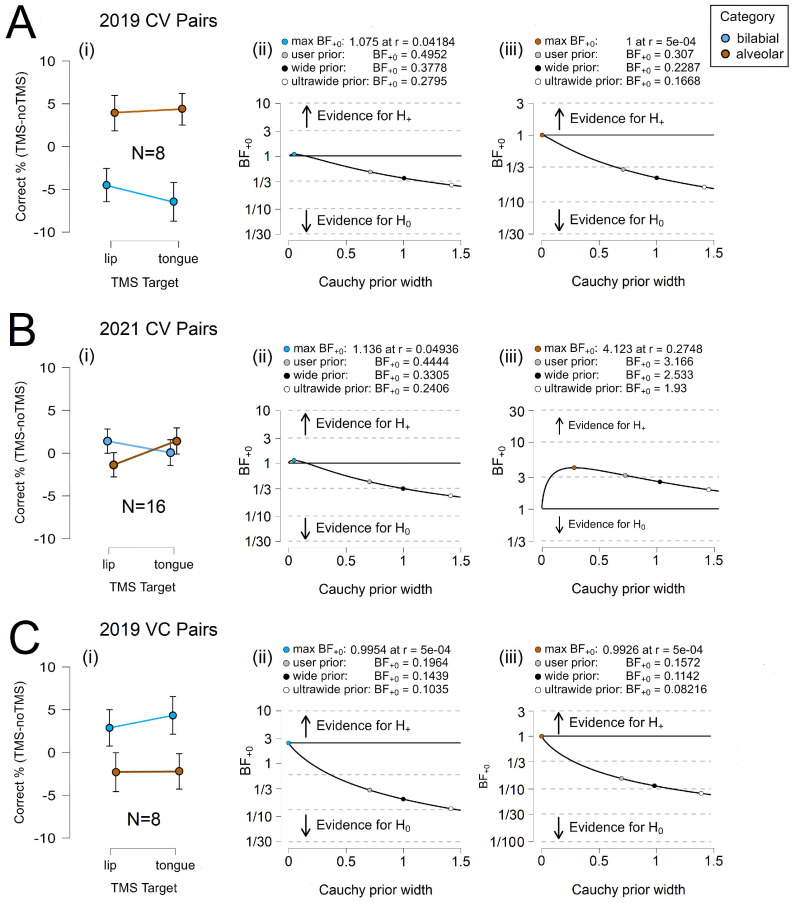
Neural Category Decoding. (**A**) 2019 CV results for (**i**) relative accuracy and (**ii**) bilabial and (**iii**) alveolar parameter estimation. (**B**) 2021 CV results for (**i**) relative accuracy and (**ii**) bilabial and (**iii**) alveolar parameter estimation. (**C**) 2019 VC results for (**i**) relative accuracy and (**ii**) bilabial and (**iii**) alveolar parameter estimation. Error bars represent the 95% confidence intervals.

Figure 5 illustrates relative task accuracy and the results for parameter estimation. For CV trials, we only observed a clear dissociation between the category groups in 2021, when more participants were recruited, increasing the overall power. A double dissociation was not visible in any of the 2019 data. As indicated in Table 3, the findings from the 2021 data appear to be largely driven by alveolar phonemes. The robustness of the Bayes factor to our prior specification is shown in BF_+0_ as a function of the prior width, *r*. The Bayes factor appears to be relatively stable across several Cauchy prior widths in this analysis.

#### 3.2.2. Individual Phonemes

We evaluated whether the effects observed at the category level would persist for individual phonemes. Whether individual phonemes would show a graded effect across TMS targets was again of particular interest, and we analyzed the 2021 CV data. In this analysis, Table 4 does reveal a graded response to stimulation, although not all phonemes responded as anticipated. The Bayes factor indicates moderate evidence for $H_{0}$ for /p/ and no evidence for $H_{1}$ for /d/ in the 2021 conditions. The BF_+0_ equaled 0.187 for /p/ and 0.638 for /d/, indicating that the data were 0.187 and 0.638 times more likely to occur under $H_{+}$ than $H_{0}$. This result was not surprising for /p/, which did not respond robustly to TMS across the experiments and analyses, presumably due to its combination of features as an unvoiced bilabial phoneme. The result for /d/, however, contradicted our assumptions that it would show the greatest increase in decoding accuracy as a voiced alveolar phoneme.

The results for the other two phonemes were similarly unexpected: the Bayes factor indicated minimal evidence for $H_{0}$ for /b/ and moderate evidence for /t/. The BF_+0_ equaled 1.071 and 3.629, respectively, indicating that the data were 1.071 and 3.629 times more likely to occur under $H_{+}$ than $H_{0}$. The error percentages for all conditions were small, with the exception of /p/. We may consider that, perhaps, the more effortful processing of unvoiced phonemes (/t/,/p/) led to increased decoding accuracy and an increased error for /p/, as it may be better described by an alternate model. However, this remains speculative and not entirely consistent with the category-level findings. We also cannot discount the limitations of the method: the coil was heavy and prone to some movement; thus, targeting may not have remained ideal throughout the entire TMS block, which became more apparent in the more specific phoneme-level analysis. Nonetheless, we did observe an effect on decoding accuracy and a graded response across phonemes.

In the VC conditions, the results were more straightforward. The Bayes factor indicated moderate evidence for $H_{0}$ across all phonemes. The BF_+0_ equaled 0.244 and 0.326 for bilabial phonemes /b/ and /p/, indicating that the data were 0.244 and 0.326 times more likely to occur under $H_{+}$ than $H_{0}$. These values closely resemble those obtained for alveolar phonemes, with the BF_+0_ equaling 0.235 and 0.244 /d/ and /t/, indicating that the data were 0.235 and 0.0244 times more likely to occur under $H_{+}$ than $H_{0}$. The error percentages remained consistently small. These findings are consistent with the assumption that phonemes in the VC condition would not be affected by TMS due to changes in the timing of TMS pulses.

**Figure 6:**
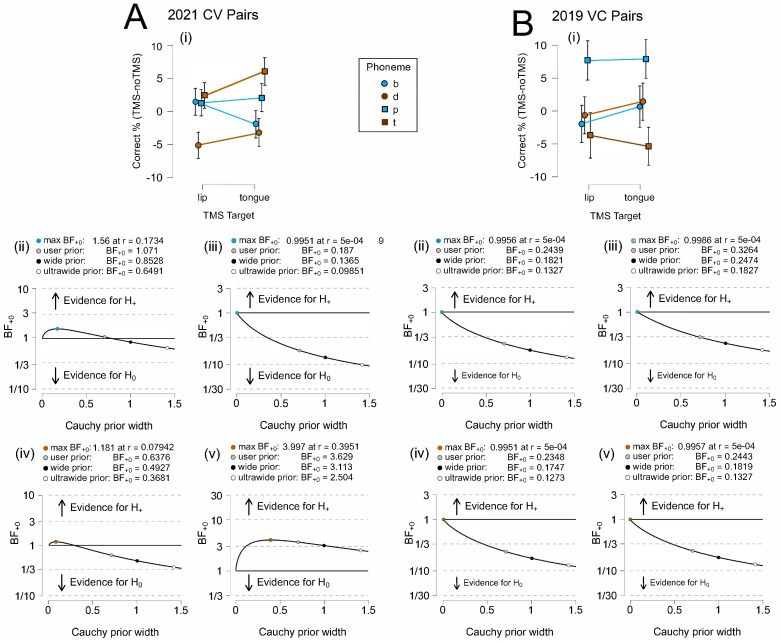
Neural Category Decoding. (**A**) 2021 CV results for (**i**) relative accuracy and robustness parameters for (**ii**) /b/, (**iii**) /p/, (**iv**) /d/, and (**v**) /t/. (**B**) 2019 VC results for (**i**) relative accuracy and robustness parameters for (**ii**) /b/, (**iii**) /p/, (**iv**) /d/, and (**v**) /t/. Error bars represent the 95% confidence intervals.

Figure 6 illustrates relative task accuracy and the results for parameter estimation. For the CV trials, despite the different states of evidentiality observed for the four phonemes, we found a double dissociation between three of the phonemes (/b/,/d/,/t/) in the 2021 CV condition. A double dissociation was not visible in any of the 2019 data. As indicated in Table 4, the findings from the 2021 data appear to have been largely driven by alveolar phonemes. The robustness of the Bayes factor to our prior specification is shown in BF_+0_ as a function of the prior width, *r*. The Bayes factor appeared to be relatively stable across several Cauchy prior widths only for /t/ in the CV condition.

In summary, the results of speech decoding were not as pronounced as those for the behavioral task. We observed consistencies between the two sets of analyses, particularly in regard to the differences seen for CV versus VC stimulus pairs. Again, the performance trended towards a more accurate perception of alveolar phonemes during TongM1 TMS for phoneme categories, and there was a greater disambiguation of individual phonemes at the TongM1 TMS target. Although, given that we are assessing relative accuracy, the target inducing a greater effect on /b/ is not clear. In the phoneme-level decoding analysis, it is less clear whether there is an additional effect of voicing at play, or a more relevant alternative factor may be the role of effortful processing. Nonetheless, it is notable that all three phonemes associated to some degree with the TongM1 TMS site trended towards an effect, and we may again conclude that not all phonemes are equally affected by the protocol.

### 3.3. Statistical Comparisons Across Data Sets

Additional inferential statistics were performed in order to better understand whether a relationship existed between the results of the phoneme discrimination task and neural decoding. We utilized the 2021 data set, given that it offered the largest participant number and a more optimal decibel level for white noise. To assess which, if any, of three factors (task accuracy, TMS target, and category or phoneme) might have been a predictor of decoding accuracy, a mixed-effects linear regression was performed. In particular, we assessed whether the accuracy achieved in the behavioral task could be considered a significant predictor of the neural decoding accuracy. The average decoding accuracy in the LipM1 TMS condition, the TongM1 TMS condition, and the two target-specific sham conditions was modeled as a function of the average task accuracy across participants, with a separate calculation for each unique combination of these factors. Participants were used as the random-effects grouping factor; however, minimal variance was observed.

The behavioral task-accuracy metrics already reflect our hypotheses regarding the association between TMS target sites and phoneme feature sets because task accuracy is assumed to vary by these factors. Therefore, we anticipated that one of the main effects of task accuracy would be a significant predictor of decoding accuracy. A significant main effect of the phoneme category or an individual phoneme would suggest that a particular category or phoneme is inherently more “marked” in terms of its neural signature in the brain. A significant effect of the target would suggest that one target is inherently better for generating perceptible signals. There was no evidence to support these additional hypotheses.

In Table 5, the relative improvement in each model over the next best-fit model was evaluated to identify which model provided the greatest explanatory value. For phoneme categories, task accuracy and target were found to be significant main effects, although only the former was highly significant. In accordance with our hypothesis, the model with the highest significance and the lowest sample variance was Decoding Accuracy∼Task Accuracy (*F*(1,135) = 11.510, *p* = 0.0009). For individual phonemes, Task Accuracy and Target were found to be the main effects, and there was a significant interaction between Task Accuracy and Phoneme. The model with the highest significance was Decoding Accuracy∼Task Accuracy * Phoneme (*F*(3,119) = 12.488, *p*≤ 0.0001). The model with the second highest significance and the lowest sample variance was Decoding Accuracy∼Task Accuracy (*F*(1,119) = 13.556, *p* = 0.0003). These findings suggest that task accuracy is a significant predictor of decoding accuracy on the phoneme-category level, as well as on the individual phoneme level, with some additional effect of individual phonemes.

## 4. Discussion

This study provides a unique contribution to the literature by demonstrating that the neuromodulatory effects of TMS on behavior are reflected in the neuronal activity measured via EEG and may be indexed using neural decoding metrics. The findings offer additional evidence for the motor theory of speech perception. While the neuroimaging literature provides a strong foundation for the theory, EEG signals can be considered a more direct measure of neuronal activity than the hemodynamic response function measured in fMRI studies, which may fail to correspond to neuromodulation-induced motor excitability [65,66]. Furthermore, the findings illustrate that the mechanisms that underlie these effects and how specific brain regions contribute to speech perception may be better addressed through the investigation of articulatory feature sets. The stimulation protocol appeared to exert a short-term effect on cortical regions associated with the place of phoneme articulation, as identified by [31]. However, better relative performance was generally observed across trials and experiments at the tongue stimulation site, suggesting the influence of additional factors. No effect was anticipated in the VC trials, yet voiced phonemes in these trials also trended toward better relative performance over the TongM1 site, indicating that regions governing the voicing feature may have been partially activated and may have exhibited an effect that perseverated into pair-final consonants. This is a prospective area for future investigation.

The study raises additional methodological and practical questions. The translational goal of the study was to investigate whether neuromodulation could facilitate non-invasive neural speech decoding from EEG signals by manipulating psychomotor activity. Brain–computer interface (BCI) devices that utilize neural signals as inputs have the potential to restore communication for patients with debilitating neuromuscular diseases, such as amyotrophic lateral sclerosis (ALS) and locked-in syndrome (LIS) [67]. While the results are promising, a number of challenges must be overcome before neuromodulation can be adopted as a realistic means of improving neural speech decoding. Certain complications are inherent to conducting a TMS experiment: TMS is known to affect neurons selectively, eliciting either an overall facilitatory or inhibitory effect based on protocol timing and the type and position of the stimulated neurons [7,8,68]. In the existing technologies, M1 excitability in response to TMS varies significantly between individuals [69,70]. These methodological barriers likely account for the individual-level variation observed in our study, such that only group-level effects were present.

Likewise, the noise and physical sensations that accompany TMS have the potential to bias individual participant responses and may impede task performance by distracting or inconveniencing susceptible participants. While sham TMS techniques exist, they are generally considered insufficient to fully mask the accompanying somatosensory effects [71]. A sham TMS coil was unavailable for this study, and therefore, the results should be interpreted in light of the fact that task performance in the control condition was objectively easier than when TMS was administered. It is the relative change in performance across TMS conditions that provides insight into the extent of facilitation that may be afforded via neuromodulation. Many, but not all, TMS conditions still showed increased performance relative to the control condition in absolute terms when TMS was paired with the corresponding phoneme or phoneme category. This hints at the potential for a much stronger absolute effect if a less distracting neuromodulation technique is adopted.

On the other hand, ensuring that participants are moderately challenged during the task may be advantageous for neural decoding. In discrimination tasks, phonemes are typically immersed in white noise, such that effort is required to perceive them. The processing of hard-to-discriminate speech sounds has been shown to increase the hemodynamic response in neuroimaging studies [25]; the number of mistakes in linguistic decision trials corresponds to an increase in functional connectivity in task-relevant brain regions [72], and the presence of an effect in neuromodulation studies has been tied to task difficulty [11,23]. The motor system may, in fact, only be engaged in response to effortful processing [73]. However, even if the psychomotor activity reported in TMS studies is an artifact of task demands, rather than a component of linguistic processing [74], this is irrelevant for a purely practical solution to speech decoding. Many brain–computer interface (BCI) paradigms orient to motor processing. The assumption that a real-world solution to speech decoding need be fully naturalistic is not warranted.

However, from a technology-development perspective, an ideal neuromodulation technique would allow for the rapid and simultaneous stimulation of multiple precise targets while excluding or reducing the somatosensory effects associated with TMS. Suitable neuromodulation technologies that address these needs continue to evolve [75]. The protocol adopted in this study illustrates the facilitation of neural speech decoding in a discrimination paradigm, whereas a BCI device will need to operate upon neural signals that are not produced in response to an external stimulus. This scenario does not preclude the use of neuromodulation for the training—of participants or algorithms—as a preliminary step in the ongoing research into neural decoding. Given the evidence of a useful effect, it is important to remain open to creative solutions to the problem of real-world neural speech decoding from non-invasive EEG signals.

## 5. Conclusions

This study reproduced the double dissociation between electromagnetic stimulation site and stimulus category that was reported by [31] during a phoneme discrimination task, and it extended the paradigm to investigate whether behavioral facilitation would predict improved neural decoding. Utilizing the EEG signals recorded from participants while they executed the discrimination task as classification inputs, we found a double dissociation between the stimulation site and phoneme-category decoding accuracy that paralleled the behavioral findings. Statistical analyses indicated that task accuracy was a significant predictor of decoding accuracy when performed on EEG signals collected during the task. These findings support the hypotheses that TMS exerts a task-relevant facilitatory effect on neuronal activity and that neural decoding metrics may serve as an index for psychomotor activity. We also investigated phoneme-level effects. While there is some evidence that a focal effect can be achieved using phonemes that possess different articulatory feature sets by targeting multiple features of the sets (e.g., place of articulation and voicing), the data remain inconclusive at this sample size. Nonetheless, task accuracy and the interaction between task accuracy and phoneme type are significant predictors of decoding accuracy.

## Figures and Tables

**Table 1:** Bayesian paired samples *t*-test—phoneme category-discrimination task.

Year	Category	Measure 1		Measure 2	BF_+0_	Error %
2019	Bilabial CV	LipM1 TMS	>	TongM1 TMS	1.188	2.349 × 10^−5^
2019	Alveolar CV	TongM1 TMS	>	LipM1 TMS	1.361	2.002 × 10^−5^
2021	Bilabial CV	LipM1 TMS	>	TongM1 TMS	1.390	1.959 × 10^−5^
2021	Alveolar CV	TongM1 TMS	>	LipM1 TMS	2.002	4.661 × 10^−5^
2019	Bilabial VC	LipM1 TMS	>	TongM1 TMS	0.121	2.441 × 10^−4^
2019	Alveolar VC	TongM1 TMS	>	LipM1 TMS	0.807	0.006

**Table 2:** Bayesian paired samples *t*-test—individual phoneme discrimination task.

Year	Category	Measure 1		Measure 2	BF_+0_	Error %
2021	B CV	LipM1 TMS	>	TongM1 TMS	3.255	2.648 × 10^−4^
2021	P CV	LipM1 TMS	>	TongM1 TMS	0.646	6.038 × 10^−7^
2021	D CV	TongM1 TMS	>	LipM1 TMS	0.596	1.212 × 10^−6^
2021	T CV	TongM1 TMS	>	LipM1 TMS	2.010	7.809 × 10^−6^
2019	B VC	LipM1 TMS	>	TongM1 TMS	0.959	0.020
2019	P VC	LipM1 TMS	>	TongM1 TMS	0.344	0.003
2019	D VC	TongM1 TMS	>	LipM1 TMS	1.385	2.875 × 10^−5^
2019	T VC	TongM1 TMS	>	LipM1 TMS	0.336	0.003

**Table 3:** Bayesian paired samples *t*-test—neural phoneme-category decoding.

Year	Category	Measure 1		Measure 2	BF_+0_	Error %
2019	Bilabial CV	LipM1 TMS	>	TongM1 TMS	0.495	1.391 × 10^−6^
2019	Alveolar CV	TongM1 TMS	>	LipM1 TMS	0.307	0.026
2021	Bilabial CV	LipM1 TMS	>	TongM1 TMS	0.444	1.230 × 10^−5^
2021	Alveolar CV	TongM1 TMS	>	LipM1 TMS	3.166	1.307 × 10^−4^
2019	Bilabial VC	LipM1 TMS	>	TongM1 TMS	0.196	5.683 × 10^−4^
2019	Alveolar VC	TongM1 TMS	>	LipM1 TMS	0.157	0.001

**Table 4:** Bayesian paired samples *t*-test—individual phoneme discrimination task.

Year	Category	Measure 1		Measure 2	BF_+0_	Error %
2021	B CV	LipM1 TMS	>	TongM1 TMS	1.071	2.521 × 10^−5^
2021	P CV	LipM1 TMS	>	TongM1 TMS	0.187	0.010
2021	D CV	TongM1 TMS	>	LipM1 TMS	0.638	6.458 × 10^−6^
2021	T CV	TongM1 TMS	>	LipM1 TMS	3.629	3.470 × 10^−5^
2019	B VC	LipM1 TMS	>	TongM1 TMS	0.244	6.211 × 10^−4^
2019	P VC	LipM1 TMS	>	TongM1 TMS	0.326	8.916 × 10^−4^
2019	D VC	TongM1 TMS	>	LipM1 TMS	0.235	6.365 × 10^−4^
2019	T VC	TongM1 TMS	>	LipM1 TMS	0.244	6.198 × 10^−4^

**Table 5:** Mixed-effects linear regression summary.

Effect	df	F	*p*-Value
Task Accuracy	1, 135	11.510	0.0009
Category	1, 135	2.257	0.1106
Target	3, 135	2.811	0.0419
Task Accuracy * Category	1, 135	0.931	0.3364
Task Accuracy * Target	3, 135	0.510	0.6762
Category * Target	3, 135	1.487	0.2210
Task Accuracy * Category * Target	3, 135	0.835	0.4771
Task Accuracy	1, 119	13.556	0.0003
Phoneme	9, 119	1.299	0.2444
Target	3, 119	3.192	0.0262
Task Accuracy * Phoneme	3, 119	12.488	<0.0001
Task Accuracy * Target	3, 119	0.093	0.9638
Phoneme * Target	9, 119	1.299	0.2444
Task Accuracy * Phoneme * Target	9, 119	0.685	0.7217

Note: Type III sum of squares.

## Data Availability

De-identified data have been deposited and are publicly available at Open Science Framework (https://osf.io/e82p9/) (accessed on 2 August 2024); the original code is available at https://github.com/mcjpedro/speech_decoding(accessed on 2 August 2024), and a detailed description of the characteristics of the data sets [76] can serve as an additional guide to the methods employed. For each trial, event timestamps are provided in .csv format, with one file for each recording session. The events include (i) the second (final) TMS pulse of the pair, (ii) the sound-stimulus onset, and (iii) the subsequent phoneme onsets. In addition to timestamps, the files provide labels for presented (true) and identified phoneme stimuli. The reproduction and sharing of this information is allowed under the CC BY 4.0 http://creativecommons.org/licenses/by-nc-nd/4.0/(accessed on 2 August 2024). All results can be found in the folders labeled Study1, Study2, or Across Studies. The raw .cnt EEG files can be read in MATLAB with the FieldTrip Toolbox [77] and with the Brainstorm [78] eepv4_read.m function, or in Python with the libeep library.

## Supplementary Materials

The following supporting information can be downloaded at: https://www.mdpi.com/article/10.3390/brainsci14090895/s1, Figure S1: All Phonemes in 2019. (**A**) Phoneme discrimination results for (**i**) CV pairs and (**ii**) individual phonemes. (**B**) Neural decoding results for (**i**) CV pairs and (**ii**) individual phonemes. These line graphs include data from both /d/ and /t/ trials. An unusually high level of correct responses in the /d/ control condition relative to the other phonemes is apparent in the downward displacement of the line representing /d/ responses along the y-axis. Error bars represent the 95% confidence intervals. Interestingly, we observed that the phonemes that obtained lower task-accuracy results showed a higher decoding accuracy. Therefore, the role of effortful processing in neural speech decoding may be a relevant area of further study.; Figure S2: Discrimination Results by Individual Phoneme. (**A**) The 2019 CV accuracy rates for (**i**) b, (**ii**) p, (**iii**) d, and (**iv**) t. (**B**) The 2021 CV accuracy rates for (**i**) b, (**ii**) p, (**iii**) d, and (**iv**) t. The difference between the percentage of correct responses in the experimental condition and its matched control condition was within approximately 5% for all phonemes, with the exception of /d/ in 2019, which showed a difference between the experimental and control condition that was approximately twice as large and marginally significant or significant. The decibel level of the white noise in which the stimuli were immersed was increased in 2021 to correct this discrepancy. Error bars represent the 95% confidence intervals. Note: Here, we provide the aggregate count of correct trials for all participants. Other figures in this paper calculate relative percentages on the subject level, rather than the group level, to allow for statistical analysis. Some discrepancies may, therefore, be observed between the two visualizations, as they do not strictly represent the same information. The variation in subject-level means may be reflected in the error bars for the mean of the entire data set. ~ *p* < 0.10; * *p* < 0.05.
