# Supplementary material for: Transcranial Magnetic Stimulation Facilitates Neural Speech Decoding
Source: Brain Sci. 2024 Sep 2;14(9):895. doi: 10.3390/brainsci14090895 (PMC11430724; doi:10.3390/brainsci14090895)
Supplement: Supplementary file 1 [file brainsci-14-00895-s001.zip › brainsci-3107153-supplementary.pdf]

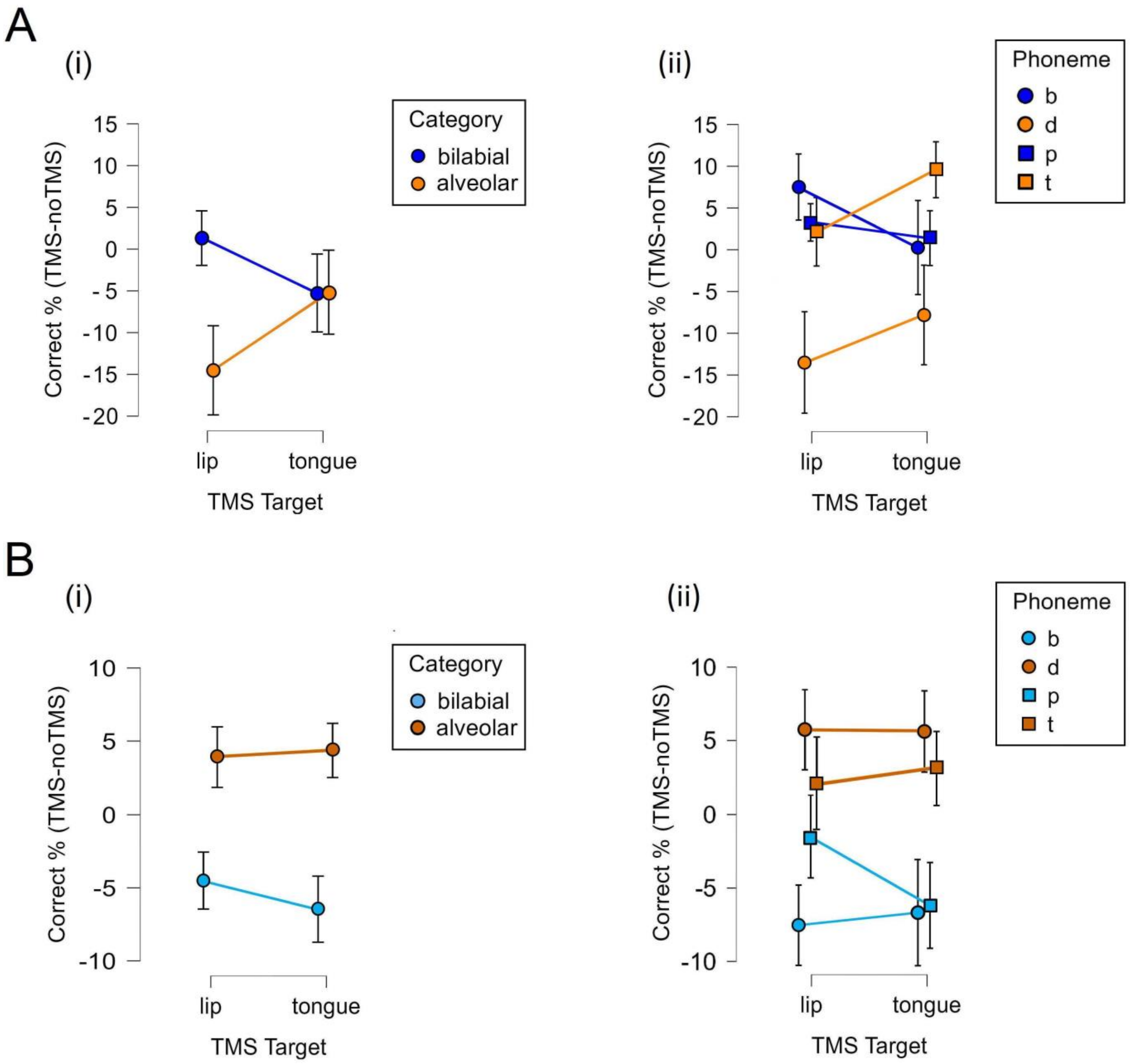

Figure S1. All Phonemes in 2019. (**A**) Phoneme discrimination results for (**i**) CV pairs and. (**ii**) individual phonemes. (**B**) Neural decoding results for (**i**) CV pairs and (**ii**) individual phonemes. These line graphs include data from both /d/ and /t/ trials. An unusually high level of correct responses in the /d/ control condition relative to the other phonemes is apparent in the downward displacement of the line representing /d/ responses along the y-axis. Error bars represent the 95% confidence intervals. Interestingly, we observed that the phonemes that obtained lower task-accuracy results showed a higher decoding accuracy. Therefore, the role of effortful processing in neural speech decoding may be a relevant area of further study.

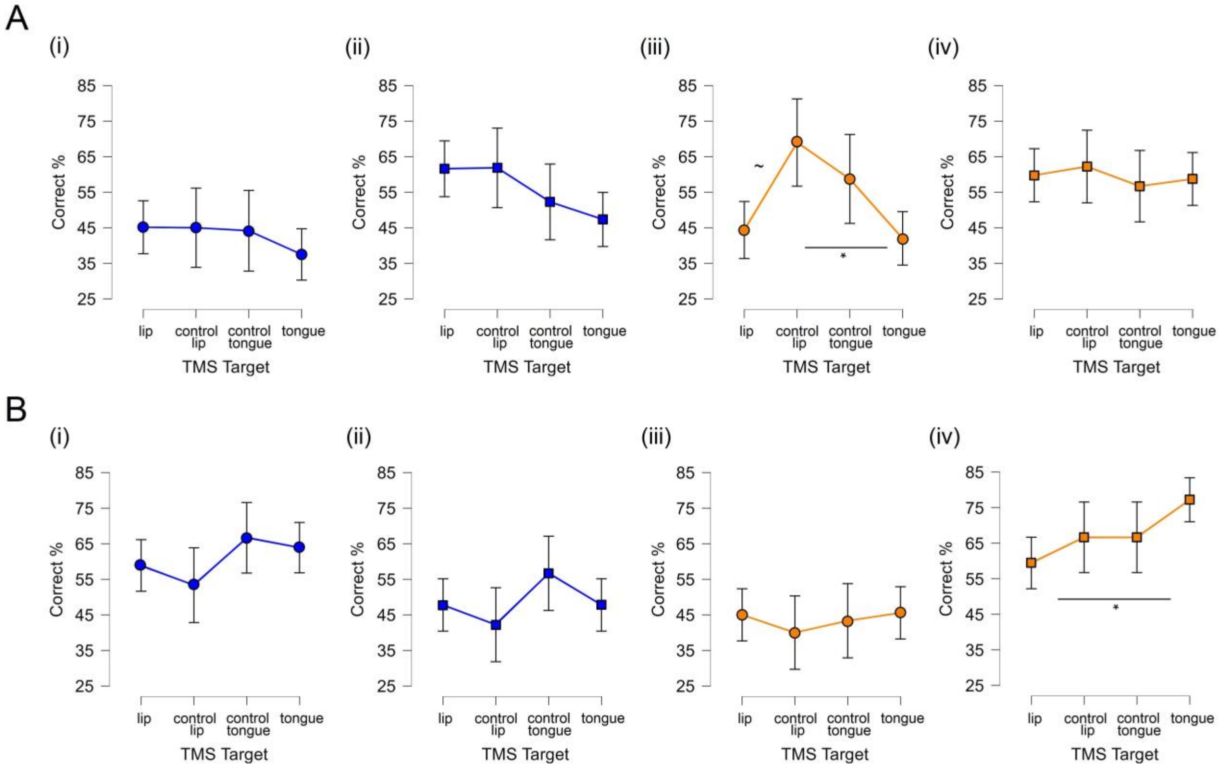

Figure S2. Discrimination Results by Individual Phoneme. (**A**) The 2019 CV accuracy rates for (**i**) b, (**ii**) p, (**iii**) d, and (**iv**) t. (**B**) The 2021 CV accuracy rates for (**i**) b, (**ii**) p, (**iii**) d, and (**iv**) t. The difference between the percentage of correct responses in the experimental condition and its matched control condition was within approximately 5% for all phonemes, with the exception of /d/ in 2019, which showed a difference between the experimental and control condition that was approximately twice as large and marginally significant or significant. The decibel level of the white noise in which the stimuli were immersed was increased in 2021 to correct this discrepancy. Error bars represent the 95% confidence intervals. Note: Here, we provide the aggregate count of correct trials for all participants. Other figures in this paper calculate relative percentages on the subject level, rather than the group level, to allow for statistical analysis. Some discrepancies may, therefore, be observed between the two visualizations, as they do not strictly represent the same information. The variation in subject-level means may be reflected in the error bars for the mean of the entire data set. ~ $p < 0.10$;.* $p < 0.05$.
